# Comparative analysis of the accelerated aged seed transcriptome profiles of two maize chromosome segment substitution lines

**DOI:** 10.1371/journal.pone.0216977

**Published:** 2019-11-11

**Authors:** Li Li, Feng Wang, Xuhui Li, Yixuan Peng, Hongwei Zhang, Stefan Hey, Guoying Wang, Jianhua Wang, Riliang Gu

**Affiliations:** 1 Seed Science and Technology Research Center, Beijing Innovation Center for Seed Technology (MOA), Beijing Key Laboratory for Crop Genetic Improvement, College of Agronomy and Biotechnology, China Agricultural University, Beijing, China; 2 Institute of Crop Sciences, Chinese Academy of Agricultural Sciences, Beijing, China; 3 Department of Agronomy, Iowa State University, Ames, Iowa, United States of America; ICAR-Indian Institute of Agricultural Biotechnology, INDIA

## Abstract

Seed longevity is one of the most essential characteristics of seed quality. Two chromosome segment substitution lines, I178 and X178, which show significant differences in seed longevity, were subjected to transcriptome sequencing before and after five days of accelerated aging (AA) treatments. Compared to the non-aging treatment, 286 and 220 differentially expressed genes (DEGs) were identified after 5 days of aging treatment in I178 and X178, respectively. Of these DEGs, 98 were detected in both I178 and X178, which were enriched in Gene Ontology (GO) terms of the cellular component of the nuclear part, intracellular part, organelle and membrane. Only 86 commonly downregulated genes were enriched in GO terms of the carbohydrate derivative catabolic process. Additionally, transcriptome analysis of alternative splicing (AS) events in I178 and X178 showed that 63.6% of transcript isoforms occurred AS in all samples, and only 1.6% of transcript isoforms contained 169 genes that exhibited aging-specific AS arising after aging treatment. Combined with the reported QTL mapping result, 7 DEGs exhibited AS after aging treatment, and 13 DEGs in mapping interval were potential candidates that were directly or indirectly related to seed longevity.

## 1. Introduction

Aging is an inevitable process affecting seed longevity in plants. Seed longevity is the length of time that a seed remains viable, which is accompanied by a progressive loss of quality or viability over time. The loss of seed longevity greatly depends on seed moisture and the relative humidity and temperature of storage conditions **[1, 2, 3].** Reactive oxygen species (ROS) accumulate during seed aging, and plant mitochondria rely on the ascorbate and glutathione (AsA-GSH) cycle to scavenge ROS. During seed storage, the activity of the AsA-GSH cycle is reduced, which results in ROS accumulation **[4, 5].** Furthermore, a previous study found that ROS accumulation also results in a decreased ability to utilize seed storage proteins (SSPs) in seeds, of which the carbonylation of SSPs was observed in aged seeds **[6, 7]**. High concentrations of H_2_O_2_, malondialdehyde (MDA), and end-products of lipid peroxidation were considered critical factors contributing to seed aging or deterioration, consequently influencing seed longevity and vigor **[8–11]**. In addition, the activity of mitochondria in seeds is significantly decreased during long-term storage, resulting in a decrease of seed vigor and inhibition of seed germination **[5, 12]**. The level of antioxidants and energy supply are also important factors in regulating seed longevity.

The molecular mechanism of crop seed longevity is still far behind the other important agronomics traits. Approaches of omics have been used to investigate protein expression patterns during seed storage in the model plant Arabidopsis and some crops **[13, 3, 14, 15, 7, 16]**. Sano et al. performed an RNA-seq experiment on bulked RILs of Est-1 × Col-0 in Arabidopsis and found that brassinosteroids (BR) are important hormones for regulating seed longevity, partially through regulating seed coat permeability **[13]**. Proteomics analysis on artificially aged wheat seeds revealed that differentially expressed proteins (DEPs) were primarily involved in metabolism, energy supply, and defense/stress responses **[14]**. GO terms related to ribosomes were enriched in aging upregulated proteins. In contrast, GO-term enrichment of aging downregulated proteins included energy supply, starch and sucrose metabolism and stress defense (ascorbate and aldarate metabolism). These findings indicate that the inability to protect against aging leads to the incremental decomposition of the stored substance via impairment of metabolism and energy supply, ultimately resulting in seed deterioration **[14]**. The characteristic of seed viability follows the reverse S-shaped survival curve during aging (includes Phases I, II and III). Comparative proteomic analysis of non-aging and 3-d aged (at a stage of transformation from Phase-I to Phase-II) rice embryos, 78 downregulated and 31 upregulated proteins were identified, with most downregulated proteins being related to energy metabolism (29%) and defense (21%), and most upregulated proteins being enriched in SSPs **[7]**. Chen et al. compared the storage ability of two wheat varieties (storage tolerant vs. storage sensitive) and found that the proteins differed notably in disease defense, protein destination and storage, and energy metabolism between two varieties. These researchers suggested that storage tolerant seeds possessed a stronger ability to activate the defense system against oxidative damage, utilizing SSPs for germination, and maintaining energy metabolism for ATP supply **[16]**. Although it was known that the gene and protein expression related to disease defense and energy metabolism was largely altered after seed harvest, the effects of these expression patterns on aging tolerance of seeds are largely unknown.

In the present study, we simulated natural seed deterioration with AA treatment on X178 and I178 (improved 178). Two chromosome segment substitution lines (CSSL) have similar genetic backgrounds but show differences in seed vigor after aging stress. Transcriptional expression analysis was performed on seeds of X78 and I178 before (0d-AA) and after 5 days of AA treatment (5d-AA) to identify DEGs after AA treatment. Furthermore, AS analysis was used to discover genes or pathways that were affected during seed aging.

## 2. Materials and methods

Maternal parent X178 is an inbred of the widely cultivated maize hybrid Nongda108 with a better agronomic performance of storability. I178 (improved X178) is derived from X178 by introgression of chromosome segments from some other inbreds **[17]**.

### Artificially accelerated aging treatment (AA)

The simultaneously freshly harvested (FH) I178 and X178 seeds were surface disinfected with 1% NaClO for five min and washed 10 times with sterile-distilled water, keeping the seeds at room temperature overnight for balancing the moisture of each sample followed by AA treatment of suspending the seeds on the metal-mesh trays, and place each tray in a closed plastic box (25×25×14 cm), then placing the plastic boxes in an aging chamber (LHC-150-11, Beijing Luxi Ltd) at 95% moisture content and 50°C temperature for 3, 5 and 7 days of different purpose. Non-accelerated aged (0d-AA) seeds were included as the control. Each treatment was represented by 3 replications. Harvested seeds were stored at 10°C.

### Zein analysis and tetrazolium chloride (TTC) staining

Samples of I178 and X178 were prepared for zein extraction, including dry seeds without any treatment (0d), seeds were imbibed in water for 6 h (0d-6h) at room temperature, seeds were germinated in prewetted crepe cellulose paper (CCP) rolls for 48 h at room temperature (0d-48h), and directly AA-treated seeds at 3 day, 5 days and 7 days (3d-AA, 5d-AA and 7d-AA) according to the above methods. The above samples of both I178 and X178 were powdered in liquid N_2,_ and 50 mg of powder was used for zein extraction **[18]**. After removing the lipids with petroleum benzin and dissolving the samples with protein extraction buffer (12.5 mM sodium borate, 2% 2-mercaptoethanol, 1% SDS and pH 10), samples were incubated at 37°C for 5 min and centrifuged at 14,800 rpm for 15 min. The supernatant containing total protein was incubated with absolute ethanol for 2 h. After centrifugation at 14,800 rpm for 15 min, the supernatant containing zein was dissolved in IPG solution (8 M urea, 220 mM DTT and 2% CHAPS) and measured with the Easy II Protein Quantitative Kit (BCA) (DQ111-01, TransGen Biotech, Beijing).

The TTC for soybean (*Glycine max*.) vigor test published by international seed testing association (ISTA) was used for detecting seed vigor after AA treatment **[19]**. Maize seeds were preimbibed in water for 20 h at room temperature, each seed was cut longitudinally through the embryo using a razor blade, and the seeds were incubated in 0.1% TTC, for 1 h and washed three times with water. The staining of the embryo was imaged using a stereomicroscope (S8APO, Leica, Germany).

### RNA-seq

One hundred seeds were ground in liquid nitrogen. Then, 0.1 g powder was used for isolating the mRNA with the RNAprep pure Plant Kit (Cat#DP432, TIANGEN, Beijing). RNA quality was checked with a 2% agarose gel. High-quality RNA was used for detecting RNA integrity number (RIN) with the Agilent Bioanalyzer 2100 (Agilent, USA). Samples with RIN 8~9 were used for RNA-Seq library preparation and sequencing on an Illumina HiSeq2500 platform (Berry Genomics, Beijing) with two biological replications. The generated raw reads were uploaded to NCBI’s SRA database and are available under the accession number PRJNA556780.

### qRT-PCR

Total RNA for the qRT-PCR experiment was extracted as described above. High-quality RNA was used for reverse transcription with the OneScript cDNA Synthesis Kit (Cat#G234, ABM, Canada). The Fast Sybr Green Master Mix (Applied Biosystems, Foster City, CA, USA) was used according to the manufacturer's instructions in a reaction volume of 10 μl. qRT-PCR was conducted on an ABI Quantstudio^™^ DX Real-Time PCR system (Applied Biosystems). PCR conditions included initial denaturation for 2 min at 95°C followed by 40 cycles of denaturation at 95°C for 30 s, hybridization at 60°C for 40 s, and elongation at 68°C for 10 s. Gene *GAPDH* was used as an internal control with the primers GAPDH-F: AGGATATCAAGAAAGCTATTAAGGC and GAPDH-R: GTAGCCCCACTCGTTGTCG **[20]**. The 2^-ΔΔct^ method was used to calculate the relative level of gene expression, and the expression in I178 dry seed was set to 1. All qRT-PCR reactions were performed with three biological replicates.

### RNA-seq data analysis

Genes were considered expressed at an FPKM value (fragments per kilobase million) > 0.1 as calculated by htseq-count in HTSeq software. To identify genes involved in seed aging, the comparison of gene expression between 0d-AA and 5d-AA was performed in both I178 and X178. DESeq2 was used for differential expression analysis with the Fold Change (FC) >1.5 and adjusted P-value (*q-value)* <0.05. A Venn diagram was generated using online software (http://bioinfogp.cnb.csic.es/tools/venny/index.html) **[21]**. Agri-Go enrichment was also performed using the online database (Agri GO v2.0; http://systemsbiology.cau.edu.cn/agriGOv2/#) **[22].** Gene organ- or tissue-specific expression levels were compared with the online q-teller database (http://www.qteller.com/qteller4/). The MapMan software was used to display differentially expressed gene sets onto diagrams of metabolic pathways and other biological processes **[23].**

## 3. Results

### 3.1 Comparison of seed storability for I178 and X178

**Fig 1:**
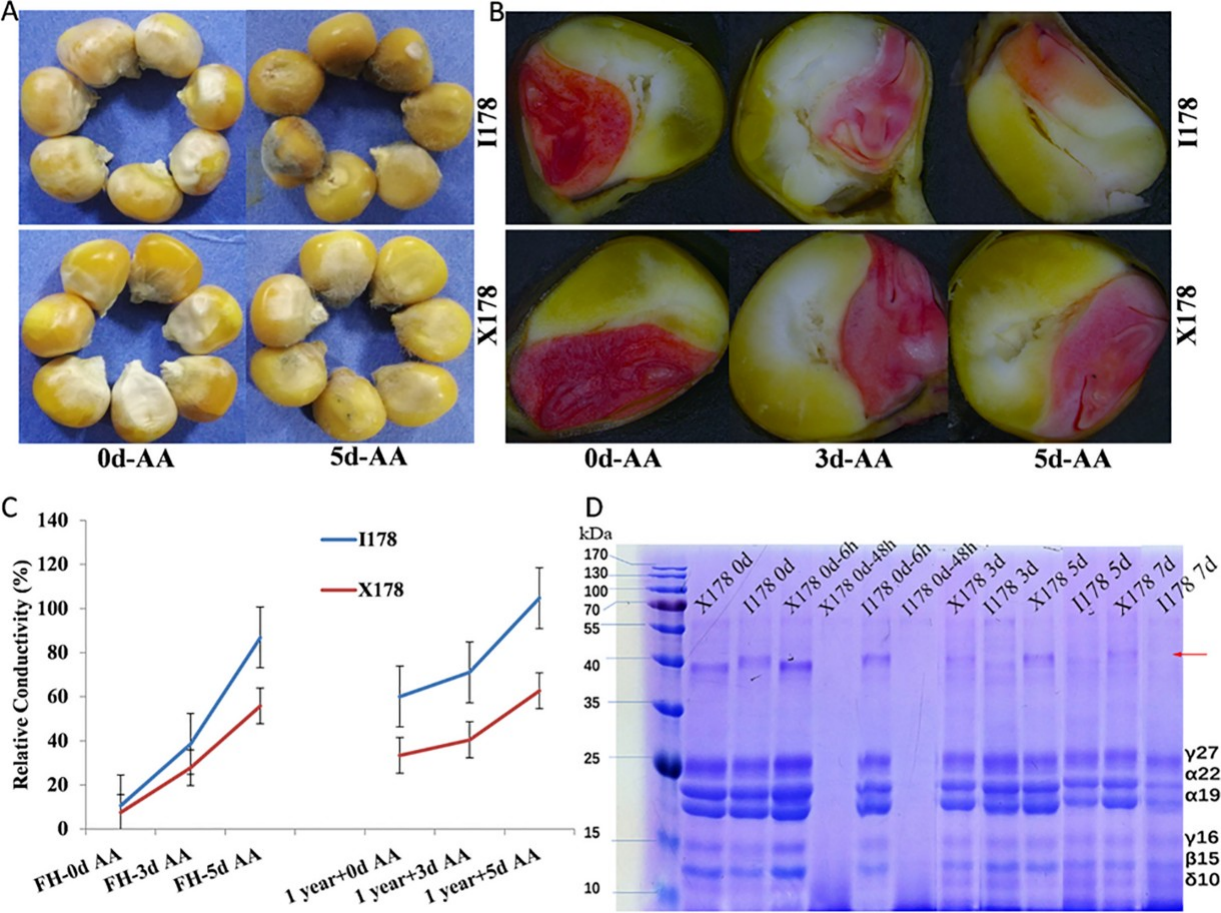
Storage characterization of I178 and X178 seed. **A).** Observation of 3d-AA and 5d-AA seeds; **B).** TTC staining of embryos at 0d-AA after 3d-AA and 5d-AA; **C).** The RC of FH seeds, one-year storage seeds treated with 3d-AA and 5d-AA; the dashed lines indicate the vigor of FH seeds after 5d-AA was lost more than that of one-year storage seeds. **D).** SDS-PAGE analysis of seeds zein, lanes 1–12: Dry seeds of X178 and I178 (X178-Dry, I178-Dry), X178 seeds after 6 h imbibed water (X178-0d-6h), 48 h germinated X178 seeds (X178-0d-48h), I178 seeds after 6 h imbibed water (I178-0d-6h), 48 h germinated I178 seeds (I178-0d-48h), X178 seeds after 3d-AA (X178-3d), I178 seeds after 3d-AA (I178-3d), X178 seeds after 5d-AA (X178-5d), I178 seeds after 5d-AA (I178-5d), X178 seeds after 77d-AA (I178-7d). The degradation of 40 kD protein is denoted by an arrow.

In terms of seed size and morphology, no significant differences could be observed between X178 and I178 due to the similar genetic background. The color of the seed coat, seed viability and vigor after long-term storage or AA treatment could reflect seed storability. More oxidization was observed in the seed coat of I178 than in that of X178 after 5d-AA treatment, which was indicated by a brown color in I178 but not in X178 (**Fig 1A**). TTC staining was used to measure seed viability. The viability was dramatically reduced in I178 but slightly reduced in X178, as the embryo stained with bright color in X178 but only stained with light color or even no color in I178 after AA treatment **(Fig 1B).** The seeds of the two lines showed a slight difference in relative conductivity (RC) before 3d-AA, but significant differences were observed after 5d-AA and one-year natural storage (**Fig 1C**). After one year of storage, the RC of I178 was two times higher than that of X178, which indicated more tolerance of X178 seed to storage than that of I178 seed **(Fig 1C)**. Comparative SDS-PAGE analysis of SSPs zein in seeds with different treatments showed that the 40 kD protein was degraded in I178 from 3d-AA, while less degradation was observed in X178. The proteins smaller than 25 kD (including γ27, α22, α19, γ16, β15 and δ10) were degraded more in I178 than in X178 after 5d-AA. All SSPs were consumed and converted into other types of proteins after 48 h of germination (**Fig 1D**).

### 3.2 Transcriptome profile of I178 and X178 seeds

**Fig 2:**
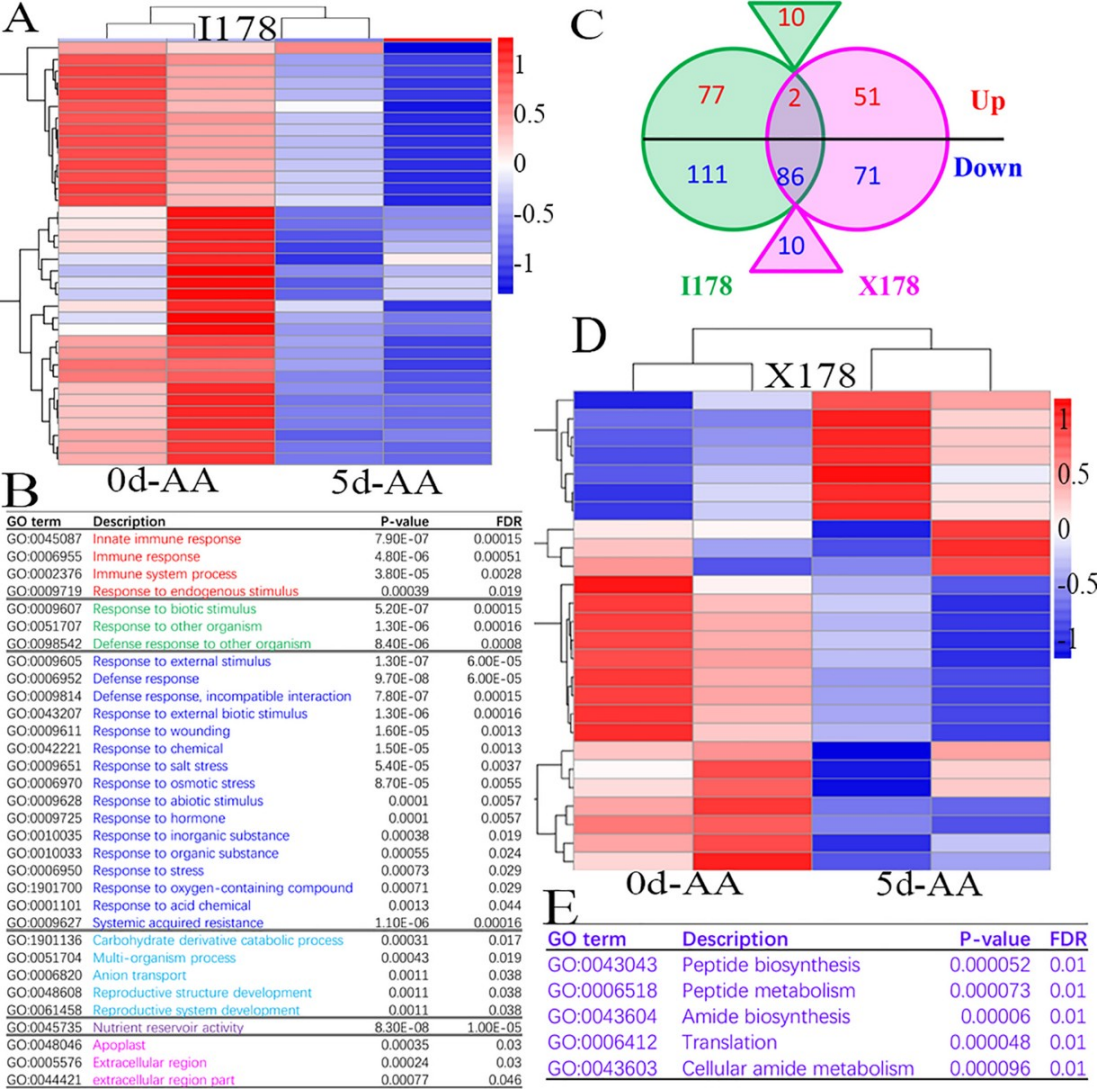
RNA-Seq of I178 and X178 and gene expression analysis before and after 5d-AA treatment. **A).** Heatmap of differentially expressed genes in I178 after 5d-AA compared with 0d-AA. **B).** Most of the significantly differentially expressed genes in I178 were enriched in 5 categories of immune system (red), biotic stress response (green), abiotic stress response (blue), carbohydrate catabolic process (light blue), nutrient reservoir activity (purple) and extracellular region (pink). **C).** Compared to the 0d-AA treatment, DEGs after 5d-AA identified in two materials with the adjusted *P*-value ≤0.05 and the FoldChange ≥1.5, there are 188 specific I178 DEGs and 122 X178 specific DEGs; among these genes, 98 common DEGs were identified in I178 and X178 with 2 common upregulated genes and 86 common downregulated genes. Ten genes were upregulated in I178, while genes downregulated in X178 were labeled in the triangle. **D).** Heatmap of DEGs in X178. **E).** GO-term enrichment of the most significantly differentially expressed genes in X178.

The cDNA libraries of the 0d-AA dry seeds and 5d-AA seeds from I178 and X178 were prepared and sequenced using an Illumina HiSeq 2500 platform. In total, 34.4~43.4 million reads with an average of 40.8 million reads were generated for each sample. At least 97.22% high quality reads were used for analysis with an average of 68.8% of the reads uniquely mapped to the B73 reference genome (ZmB73_RefGen_v3). Expression values were calculated using fragments per kilobase per million reads mapped (FPKM). In total, 92.92% of reads were mapped to protein-coding genes, and 1.36% and 5.72% were mapped to intron and intergenic regions, respectively **(S1 Table)**. Furthermore, the FPKM distribution and Pearson correlations of the two replicates ranged from 0.95–0.99 (**S1 Fig)**. In total, 26,909 and 26,514 genes were expressed (more than 20 reads, FPKM ≥0.1) in I178 and X178, respectively. Among those genes, 25,242 (more than 94%) were expressed in both seeds (FPKM > 0.1). To identify genes involved in seed aging, we focused on genes that were differentially expressed after 5d-AA (compared to 0d-AA) **(Fig 2A and 2B)**. We detected 286 and 220 DEGs in I178 and X178, respectively, with 98 DEGs detected in both I178 and X178 (log2FC ≥ 0.585), and while most of these genes were downregulated (86/98), only two upregulated genes were identified in both I178 and X178 lines. Ten genes showed diverse up- or downregulation patterns in I178 and X178. The genotype-specific DEGs detected in I178 (188 DEGs with 77 upregulated and 111 downregulated) were more than those detected in X178 (122 DEGs with 51 upregulated and 71 downregulated) (**Fig 2C and S2 Table**).

### 3.3 GO-term enrichment of aging-induced DEGs

GO terms are an internationally standardized gene function classification system for describing the properties of genes and their products in any organism, which is composed of three ontologies: biological process, cellular component, and molecular function **[22, 24]**. Based on GO-term enrichment of the 286 DEGs detected in I178, 32 GO terms were involved in five aspects, including three biological processes of the immune system, biotic stress response and abiotic stress response; two molecular functions of the carbohydrate derivative catabolic process and nutrient reservoir activity; and one cellular component of the apoplast/extracellular region and cytoplasm (**Fig 2B and S2 Fig**). For the 220 DEGs detected in X178, the GO terms were only enriched in cellular components, including organelle lumen, nucleoplasm, membrane-enclosed lumen, intracellular part and heterochromatin region, few of the DEGs were enriched in molecular function of peptide/amide biosynthesis, translation and cellular amide metabolism **(S2 Fig and Fig 2E).** GO-term enrichment of the 98 overlapping DEGs in both the I178 and X178 lines revealed functions in the cellular component of the cell part (GO:0005623, GO:0044464), membrane-enclosed lumen (GO:0031974), organelle and intracellular organelle (GO:0043226, GO:0044422, GO:0043233, GO:0043229), intracellular part (GO:0044424, GO:0070013, GO:0044446, GO:0043231), RNA polymerase (GO:0030880, GO:0000428, GO:0055029, GO:0016591), membrane bounded organelle (GO:0043227), transferase complex (GO:0061695) and nuclear part (GO:0005634, GO:0044428, GO:000319981, GO:0005654). Most of the common DEGs were downregulated in I178 and X178, and the common downregulated genes were enriched in the biological process of carbohydrate derivative catabolic and molecular function of carbohydrate derivative binding **(S2 Fig**). Only two commonly upregulated genes in I178 and X178 were found. GRMZM2G353885 encodes a TATA box binding protein (TBP)-associated factor 2, and GRMZM2G005865 has no annotation (**S2 Fig**).

### 3.4 Analysis of DEGs by qRT-PCR

**Fig 3:**
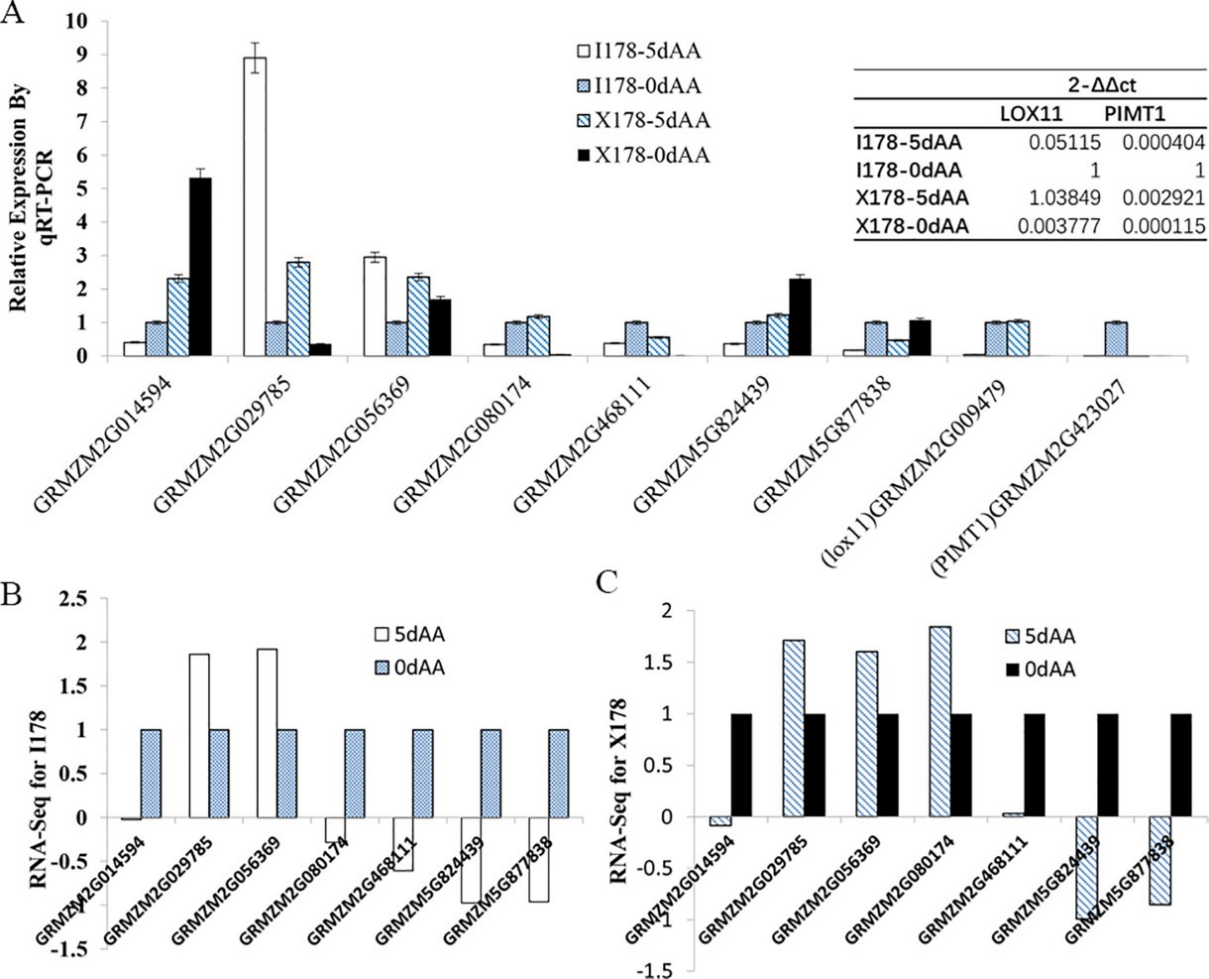
qRT-PCR validation of the RNA-Seq result. **A).** Nine genes, including 7 differentially expressed and 2 aging-related genes, were validated by qRT-PCR. RNA-Seq gene expression calculated by fold-change in I178 **(B)** and X178 **(C)**.

To validate the RNA-Seq results, 9 genes, including 7 randomly selected DEGs and 2 longevity-related genes, were selected for qRT-PCR analysis. The expression patterns of the 7 DEGs were consistent between qRT-PCR and RNA-seq, indicating that the RNA-seq gene expression was reliable. Two published aging-related genes (ZmLOX11 and ZmPIMT1) were not detected in the RNA-Seq analysis. qRT-PCR was conducted on I178 and X178 before and after 5d-AA treatment. As expected, the expression was extremely low. These results were also consistent with the q-Teller whole transcriptome expression results (http://www.qteller.com/qteller4/) **(Fig 3)**.

### 3.5 Alternative splicing (AS) analysis of aging-related transcriptions

**Fig 4:**
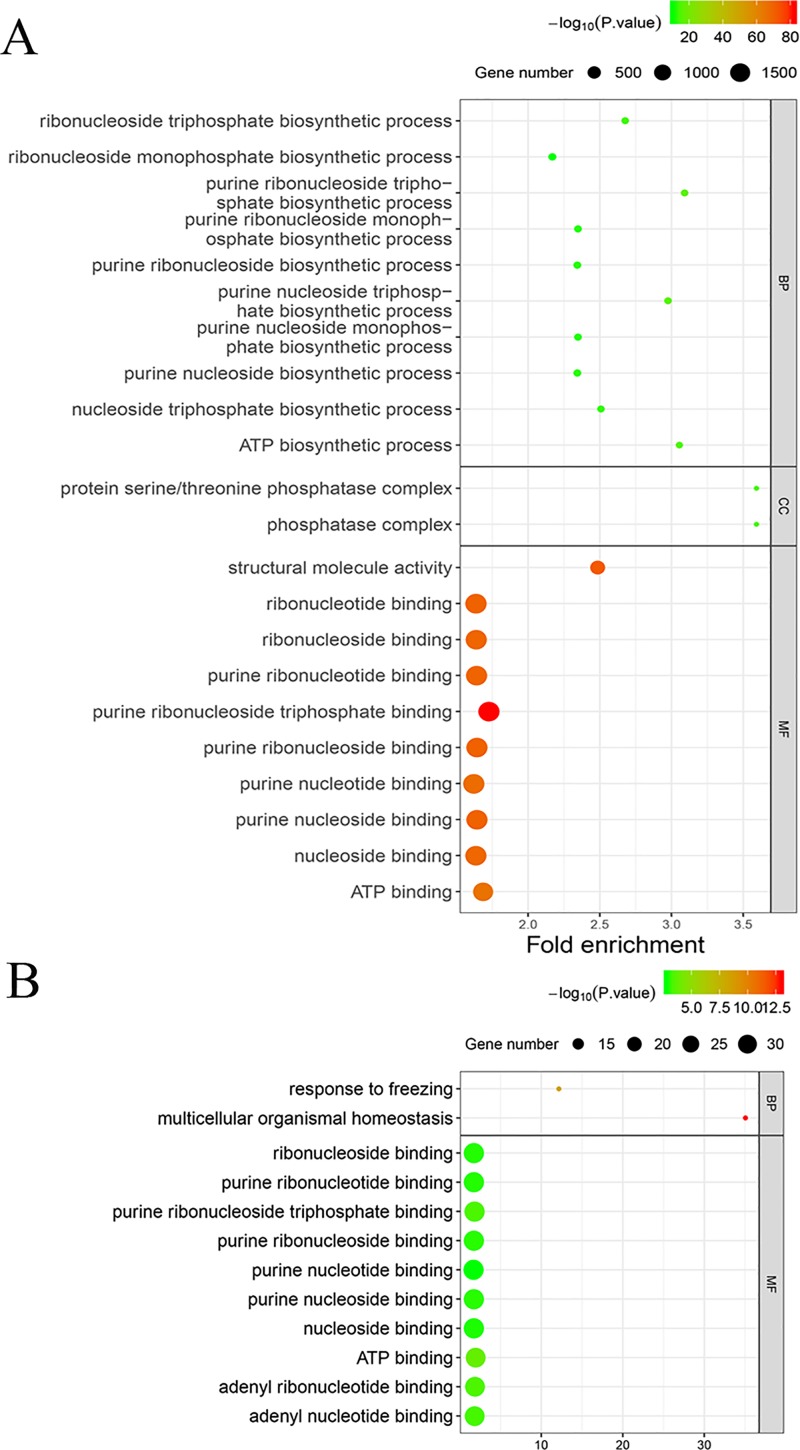
GO-term enrichment of AA induced alternative splicing genes (ASG) in I178 and X178. **A).** Biology process, cellular component and the molecular function of common ASGs in I178 and X178. **B).** Three hundred eighty-one AS genes specifically expressed after 5d-AA in I178 and X178.

In I178 and X178, we identified 51,388~59,146 AS events, including 12 different splicing types (TSS, TTS, AE, IR, MIR, MSKIP, SKIP, XIR, XMIR, XMSKIP, XSKIP), which covered 46,521~48,724 transcript isoforms (including 15,984~17,070 genes) **(S3 Table)**. Among those AS events, TSS (alternative in transcription start site) and TTS (alternative in transcription terminal site) account for 72% of the total AS events. IR (intron retention), AE (alternative exon) and SKIP (skipped exon) were also frequently observed in I178 and X178 (**S3 Fig**). In dry seeds (0d-AA), we detected 56,228 and 53,593 AS events in I178 and X178 responding for 20,446 and 19,623 transcripts, respectively. After 5d-AA, we detected 55,763 and 56,794 AS events in I178 and X178, occurring in 20,073 and 19,845 transcripts, respectively **(S3 Fig and S3 Table)**. By comparing AS before and after AA (5d-AA vs. 0d-AA) in I178 and X178, we found 63.6% transcript isoforms (15,606, cover 12,834 genes) were constitutive AS that occurred in both I178 and X178 before and after 5d-AA treatment. To further examine the role of AS genes during seed aging, we focused on AS genes uniquely identified in I178 and X178 after 5d-AA. We identified AS in 381 transcript isoforms (comprising 169 genes) in both lines. While we found AS in 849 transcript isoforms (comprising 415 genes) specifically in I178, X178 showed only 760 transcript isoforms (comprising 343 genes) after 5d-AA (**S3 Fig**). Aging-related AS genes were analyzed using Agri-Go analysis. AS genes found in both I178 and X178 showed enriched GO terms related to nucleotide biosynthesis **(Fig 4A)**. For those AS genes specifically occurring in I178 and X178 after 5d-AA, as well as such molecular functions as ribonucleoside binding and ATP binding, some were also enriched in the freezing response **(Fig 4B)**. Combined with the DEG and AS data in this study, there are only six X178-specific DEGs with AS and one I178-specific DEG with AS after 5d-AA (**Table 1 and S3 Fig**).

**Table 1:** Potential seed aging related genes.

DEG ID	Gene ID	I178_	X178_	Up/	Source.	Homologs	Annotation
		log2FC	log2FC	Down			
DEG1	GRMZM2G058970	0.87	0.86	down	**QTL**	At5G49930.1	Zinc knuckle (CCHC-type) family protein
DEG2	GRMZM2G302913	1	0.67	down	**(Chr3:8–205 Mb)**	At1G22060.1	-
DEG3	GRMZM2G358618	0.74	0.84	down		At4G03200.1	Catalytics
DEG4	GRMZM2G375807	1.03	0.88	down		At4G39850.3	Peroxisomal ABC transporter 1
DEG5	GRMZM2G379913	0.98	0.8	down		At3G06530.1	ARM repeat superfamily protein
DEG6	GRMZM2G379929	0.96	0.88	down		At3G06530.2	ARM repeat superfamily protein
DEG7	GRMZM2G438938	0.86	0.86	down		At5G02310.1	Proteolysis 6
DEG8	GRMZM5G867767	0.97	0.7	down		At3G10650.1	-
DEG9	GRMZM2G181135	1.86	0.89	down		At1G34060.1	Pyridoxal phosphate (PLP)-dependent Transferases superfamily protein
DEG10	GRMZM5G800586	1.27	0.77	down		At5G25610.1	BURP domain-containing protein
DEG11	GRMZM5G877838	0.97	0.89	down		Os01g56780.1	Plus-3 domain containing protein
DEG12	GRMZM2G074604	0.8	0.86	down	**QTL**	AT2G37040.1	PHE ammonia lyase 1
DEG13	GRMZM5G824439	0.98	0.99	down	**(Chr5:185–205 Mb)**	AT1G23230.2	-
DEG14	GRMZM2G158232	-0.98	-0.03	up	**I178_AS-DEG**	At1G53540.1	HSP20-like chaperones superfamily protein
DEG15	GRMZM5G804358	-0.13	-1.02	up	**X178_AS-DEG**	AtMG00580.1	NADH dehydrogenase subunit 4
DEG16	GRMZM2G476810	0.52	0.64	down		At5G40480.1	Embryo defective 3012
DEG17	GRMZM2G461586	-0.31	-0.78	up		At3G02260.1	Auxin transport protein (BIG)
DEG18	GRMZM2G311182	-0.39	-0.71	up		At4G02280.1	Sucrose synthase 3
DEG19	GRMZM2G138727	-0.54	-0.63	up		-	27-kDa zein protein
DEG20	GRMZM2G417682	0.69	0.61	down		-	-

Note: Thirteen DEGs in both I178 and X178 were located in QTL interval derived from RILs and F_2:3_ populations of I178 × X178 (DEG1-13); 7 AS-DEGs were identified in I178 (DEG14) and X178 (DEG15-20) after 5d-AA, were possible the Aging related candidates.

## 4. Discussion

### 4.1 Seed storability decreased dramatically in I178 after AA treatment

I178 was derived from X178 by introgression of small parts of chromosome segments from relative aging-sensitive lines. Introgressions mainly occurred on chromosomes 7 and 10 **[17]**. Both I178 and X178 showed similar agronomic performances, except the seed germinability after AA treatment **[17]**. The difference in seed vigor between X178 and I178 was further confirmed in this work by electrolytic conductivity assay, TTC staining and SDS-PAGE analysis **(Fig 1)**. In addition, the storability of X178 and I178 seeds was tested under natural storage conditions, and a slow and slight reduction of seed vigor was observed in X178 seeds compared to I178 seeds. This observation is consistent with the AA results **(Fig 1C)**.

SSPs have been described as the primary targets for oxidation in seeds. Zein comprises approximately 60% of SSPs **[25].** Carbonylation of SSPs during long-term storage is an irreversible oxidation process leading to protein deterioration in both after-ripening seeds and aged seeds **[26, 27]**. More studies found that SSPs were degraded during seed germination, which were also differentially expressed in aged seeds, indicating that SSPs play important roles in seed longevity **[28, 6, 7, 16]**. In this study, we also observed the degradation of zein after 48 h of germination in both I178 and X178 (**Fig 1D**). Previously, it has been reported that oxidative SSPs were more easily degraded into smaller polypeptides or amino acids **[16]**. Consistent with our result, the total amount of zein was more degraded in I178 than the same period of AA treatment in X178 seeds, further suggesting the important role of SSPs during seed aging.

### 4.2 Seed aging affects numerous biological processes, including stress response and carbohydrate metabolism

Aging is an inevitable process that occurs in all living organisms. The consensus molecular mechanism of aging includes the following: 1) peroxidation of the plasma membrane and disintegration of membrane system structures; 2) variation of biomacromolecules, including variation of nucleic acid (RNA and DNA) and enzyme/proteins; and 3) accumulation of toxic substances, i.e., ROS, MDA and the byproducts of seed’s physiological activity, such as alcohols and free fatty acids **[29, 16]**. In this study, 88% (86/98) of the common DEGs detected in 5d-AA-treated seeds for both I178 and X178 were downregulated. The enriched GO terms of this gene set in the biological functions category were carbohydrate derivative catabolic processes, and the molecular function category was carbohydrate derivative binding **(S2 Fig)**. We also noticed that I178, the aging-sensitive line, was more influenced during aging, of which the immune system, stress response system and the energy/nutrients supply system were all affected after 5d-AA **(Fig 1B)**. These data are in line with observations by Lv et al.; GO-terms of carbohydrate derivative pathway and stress response process were also enriched in aged wheat seeds. Interestingly, the carbohydrate derivative was enriched among the upregulated genes in aged seeds, while the stress response was enriched in the downregulated genes **[14]**. The different expression patterns of carbohydrate derivative-related genes detected in wheat and maize seeds could be explained as follows: 1) Different mechanisms of aging process between wheat and maize. 2) The different aging levels between these two experiments, by which the germination rate of aged wheat seeds was lower than 20% in Lv’s study, while it was 20% - 80% in aged I178 and X178 seeds based on Liu’s study **[17, 14].** In addition, we found 10 genes that were upregulated in I178 but downregulated in X178 after 5d-AA. These genes include the two wound-responsive family genes GRMZM2G106413 and GRMZM2G106445, one dehydration-induced gene (*ERD15*, GRMZM2G037189), and a glyceraldehyde-3-phosphate dehydrogenase C2 (*GAPC2*) gene GRMZM2G176307, which are related to carbohydrate catabolic processes. The regulation pattern of the carbohydrate catabolic process-related genes in X178 was inconsistent with Lv’s result in wheat. It is possible that the aging-resistant X178 line exhibits a different response system to aging. Alternatively, the DNA and protein repair systems may be affected in X178 compared to I178 in some certain pathways.

### 4.3 ZmPIMT1 and LOX11 were downregulated after 5d-AA

To date, several genes related to longevity or aging have been cloned **[29–32]**, including protein-L-isoaspartyl (d-aspartyl) O-methyltransferase (PIMT), a typical protein repairing methyltransferase related to seed longevity by catalyzing the transfer of a methyl group from S-adenosyl methionine to the free a-carboxyl group of abnormal L-isoAsp residues **[30, 31].** In maize, there are two orthologs of *AtPIMT* (*ZmPIMT1* and *ZmPIMT2*), and only *ZmPIMT2* was expressed. However, the expression of this gene was unchanged after 5d-AA in both the I178 and X178 lines according to our RNA-Seq results. qRT-PCR of *ZmPIMT1* showed an extremely low expression signal in all investigated samples (**Fig 3),** indicating the spatiotemporal expression specificity of *PIMT1* in dicotyledon and monocotyledon plants.

Lipoxygenase (LOX) is also a typical longevity-related protein that is involved in lipid oxidation in seeds or other tissues **[32]**. To date, 13 *ZmLOX* genes have been identified in maize, but few of them have been cloned or further characterized at the molecular level **[31]**. In Arabidopsis, *LOX2* is essential for the formation of green leaf volatiles and five-carbon volatiles **[33].** *ZmLOX11* (GRMZM2G009479), a homolog of *AtLOX2* in maize, was not expressed in this RNA-Seq experiment, which was consistent with the gene expression data in the q-Teller dataset (https://qteller.maizegdb.org/). qRT-PCR revealed low expression of *ZmLOX11* in both I178 and X178 seeds. While *ZmLOX11* was downregulated in I178, it was upregulated in X178 after 5d-AA treatment. One possibility is that *LOX11* was not specifically expressed in seeds or the spatiotemporal specificity of *LOX11* in different tissues other than seeds **(Fig 3)**.

### 4.4 Excavating genes potentially associated with seed longevity

Numerous studies have reported that seed longevity-related genes might be involved in switching off metabolic activity in seeds, repairing systems during seed imbibition, and repairing DNA, RNA or protein during stress **[29]**. Preliminary results of seed aging-related QTL mapping were performed on RILs and F_2:3_ populations of I178 × X178. Seventeen QTLs located on 5 chromosomes were identified **[17]**. To excavate genes related to seed longevity, DEGs that fall within the 17 QTL mapping intervals were selected for analysis. In total, we found 13 DEGs located in QTL intervals of chromosome 3 (11 genes) and chromosome 5 (2 genes). Of these DEGs, 10 have annotation information. DEG4 encodes a peroxisomal ABC transporter 1. In Arabidopsis, DEG4 has been shown to be essential for transporting hydrophobic fatty acids and large cofactor molecules (carrier for ATP, NAD and CoA) and plays an indispensable role in fatty acid β-oxidation, photorespiration, and degradation of ROS **[34].** It is possible that during seed aging, the ROS accumulated in seed, which was detrimental to the cellular activities and consequently affected the regulation of DEG4. DEG5 and DEG6, encoding two ARM repeat proteins, have been suggested to affect seed longevity through the ubiquitination pathway **[35]**. DEG7 encodes a protease 6. The Arabidopsis homolog *ASPG1* affected seed longevity and germination by proteolysis **[36]**. DEG10 encodes a BURP domain-containing protein, which is a newly identified protein unique to plants. BURP domain-containing proteins play an important role in plant abiotic stresses, development and metabolism via regulating the level of diverse proteins **[37, 38].** DEG12 encodes a phenylalanine ammonia lyase homolog1 (PAL1), which has been reported to be involved in multiple biological processes, including response to environmental stress **[39].** Based on the annotation and reported functions, the above genes are potential candidates for aging-induced defense responses, energy metabolism and DNA/RNA and protein repair systems **(Table 1)**.

AS is a process whereby multiple functionally distinct transcripts are encoded by a single gene. Varying transcripts are obtained by the selective removal or retention of exons and/or introns from the maturing RNA **[40, 41],** which is common in many eukaryote lineages **[42–46]**. In this study, we identified seven AS-DEGs in I178 and X178. DEG14 encodes an HSP20-like chaperone superfamily protein. DEG15 encodes an NADH dehydrogenase subunit 4, an important enzyme in the electron-transport chain in mitochondria **[47]**. DEG16 encodes an embryo defective 3012. DEG17 is an auxin transport protein (BIG) in charge of auxin polar transportation and distribution in seeds **[48, 49].** DEG18 encodes a sucrose synthase 3, which is involved in respiration **[50].** DEG19 encodes a 27-kDa zein protein (zp27), which is specifically expressed in maize and may function as a protease inhibitor **[51].** The upregulation of DEG14, 15, 17, 18 and 19 suggests that these genes play a role in seed aging by regulating such parameters as seed stress response, energy metabolism, and development.

In conclusion, this work compared the storability of two maize genotypes (I178 and X178) that had been treated with 5d-AA. A comparison of the I178 and X178 lines in terms of seed viability revealed that X178 was a storage-tolerant genotype. We analyzed the gene expression patterns of the two lines using transcriptome sequencing, a comparison of genes after 5d-AA to 0d-AA revealed that most of aging related genes were involved in defense system and carbohydrate derivative catabolic process; while compared the gene expression patterns of the two lines via Mapman enrichment, a model for the regulatory effect of gene expression on seed storage tolerance is proposed in **S4 Fig**, the differences of aging treatment between I178 and X178 were mainly reflected in energy, DNA/RNA and protein repair system, glycometabolism and defense systems. Based on our findings, seed storage tolerance is enhanced if the seeds could possess a stronger ability to (1) activate the defense system for preventing oxidative damage; (2) utilize SSPs for germination and to repair the damage; and (3) maintain energy metabolism for supplying ATP.

## Supporting information

S1 Fig Data quality demonstration of RNA-Seq. **A).** FPKM distribution of 2 replications for I178 and X178. **B).** Pearson correlation analysis between samples of I178 and X178.
(JPG)

S2 Fig GO-term enrichment analysis of the DEGs. Biology process, cellular component and the molecular function of 286 I178 DEGs **(A)** and 220 X178 DEGs **(B); C).** Only 2 co-upregulated genes were detected in both I178 and X178, one of which encodes a TBP-associated factor; **D).** A total of 98 common DEGs were identified in both I178 and X178; **E).** 86 I178 and X178 commonly downregulated genes identified in this study, most of the genes involved in carbohydrate catabolic process and carbohydrate derivative binding.
(JPG)

S3 Fig AS, ASG and AS-DEGs identified in both I178 and X178. **A).** Twelve types of AS identified in I178 and X178 after 0d-AA and 5d-AA. **B).** Transcript isoforms (and the covered genes in brackets) that occurred AS in I178 and X178 before treatment (0d-AA) and after 5d-AA, the red colored are genes specifically spliced in both I178 and X178 after 5d-AA. **C).** Alternatively, spliced DEGs in I178 and X178 after 5d-AA. Six and one AS-DEGs were identified specifically in X178 and I178, respectively.
(JPG)

S4 Fig Main metabolic pathways involved in maize I178 and X178 seed aging. Gene abundance is displayed in the colored box in red (upregulation) and blue (downregulation); the corresponding pathway is marked in blue arrow and red arrow.
(JPG)

S1 Table Number of reads sequenced and mapped to the maize genome. (XLSX)

S2 Table Expression of 98 common DEGs and the related biology process classification. (XLSX)

S3 Table Alternative splicing events and the genes involved in maize I178 and X178 seed aging. (XLSX)
