# Supplementary figures and images for: Comparative analysis of the accelerated aged seed transcriptome profiles of two maize chromosome segment substitution lines
Source: PLoS One. 2019 Nov 11;14(11):e0216977. doi: 10.1371/journal.pone.0216977 (PMC6844465; doi:10.1371/journal.pone.0216977)

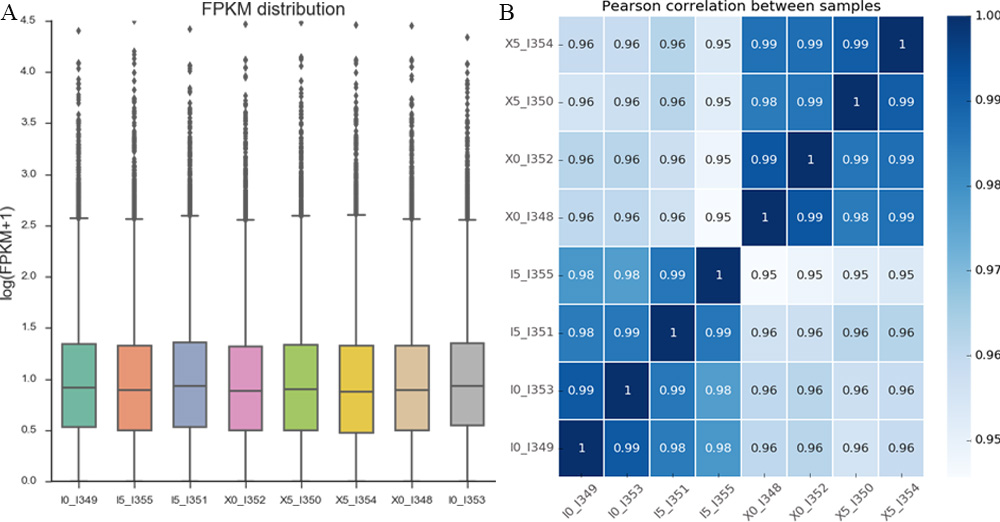

Supplement: S1 Fig — A). FPKM distribution of 2 replications for I178 and X178. B). Pearson correlation analysis between samples of I178 and X178. (JPG) [file pone.0216977.s001.jpg]

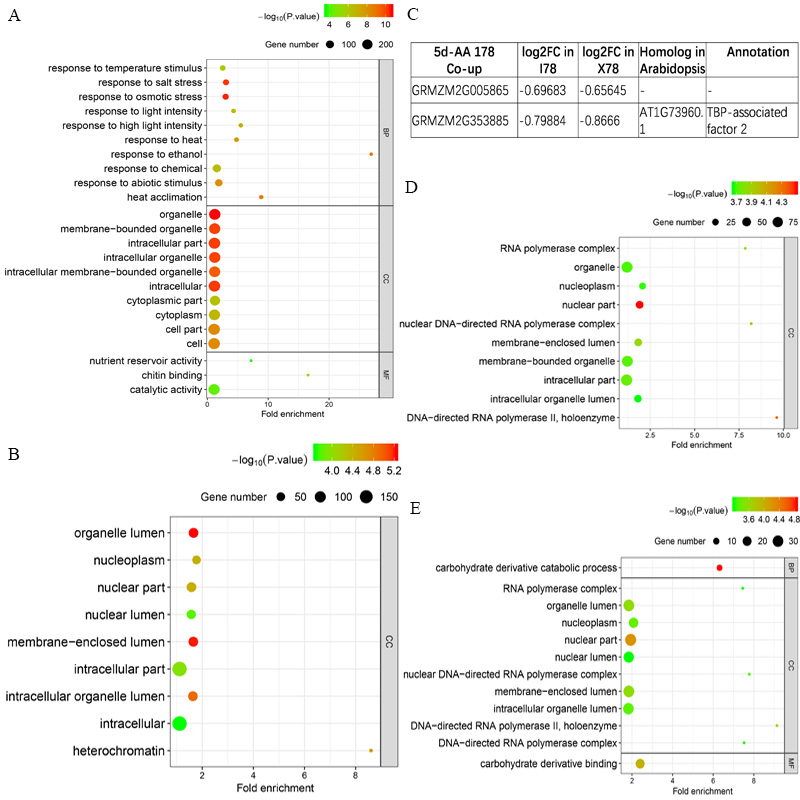

Supplement: S2 Fig — Biology process, cellular component and the molecular function of 286 I178 DEGs (A) and 220 X178 DEGs (B); C). Only 2 co-upregulated genes were detected in both I178 and X178, one of which encodes a TBP-associated factor; D). A total of 98 common DEGs were identified in both I178 and X178; E). 86 I178 and X178 commonly downregulated genes identified in this study, most of the genes involved in carbohydrate catabolic process and carbohydrate derivative binding. (JPG) [file pone.0216977.s002.jpg]

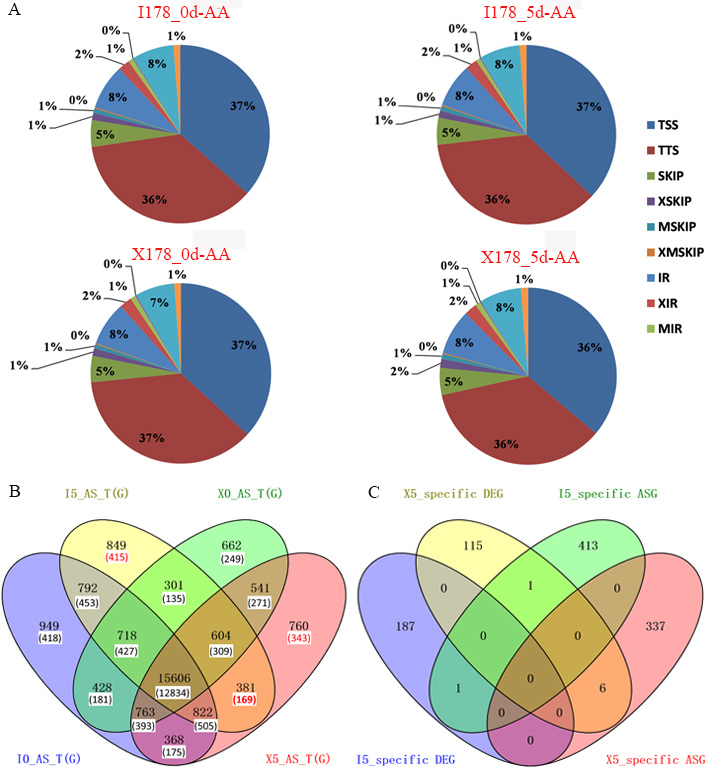

Supplement: S3 Fig — A). Twelve types of AS identified in I178 and X178 after 0d-AA and 5d-AA. B). Transcript isoforms (and the covered genes in brackets) that occurred AS in I178 and X178 before treatment (0d-AA) and after 5d-AA, the red colored are genes specifically spliced in both I178 and X178 after 5d-AA. C). Alternatively, spliced DEGs in I178 and X178 after 5d-AA. Six and one AS-DEGs were identified specifically in X178 and I178, respectively. (JPG) [file pone.0216977.s003.jpg]

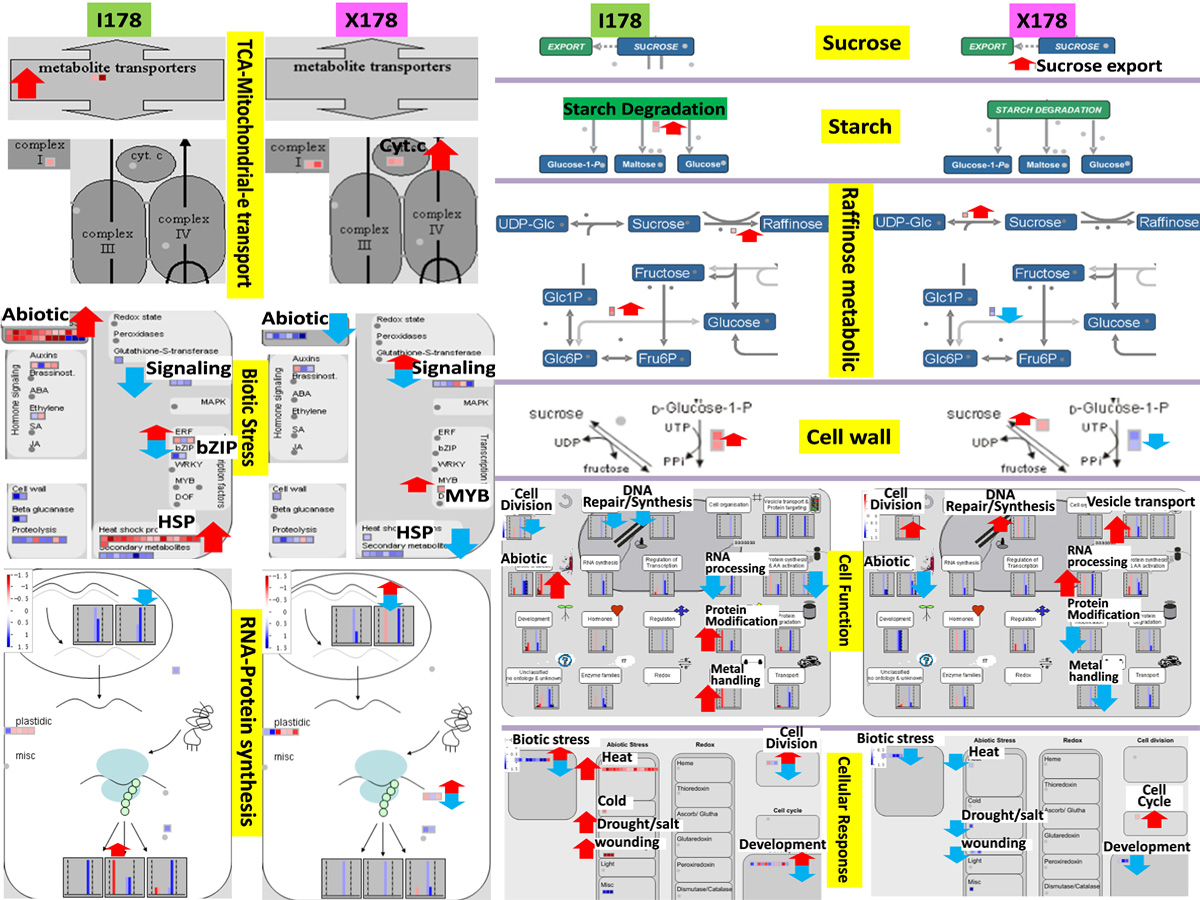

Supplement: S4 Fig — Gene abundance is displayed in the colored box in red (upregulation) and blue (downregulation); the corresponding pathway is marked in blue arrow and red arrow. (JPG) [file pone.0216977.s004.jpg]
